# Stathmin is overexpressed and regulated by mutant p53 in oral squamous cell carcinoma

**DOI:** 10.1186/s13046-017-0575-4

**Published:** 2017-08-14

**Authors:** Hai-long Ma, Shu-fang Jin, Wu-tong Ju, Yong Fu, Yao-yao Tu, Li-zhen Wang, Jiang-Li, Zhi-yuan Zhang, Lai-ping Zhong

**Affiliations:** 1 Department of Oral & Maxillofacial-Head & Neck Oncology, Ninth People’s Hospital, Shanghai Jiao Tong University School of Medicine, No 639, Zhizaoju Rd, Shanghai, 200011 China; 2 Department of Oral Pathology, Ninth People’s Hospital, Shanghai Jiao Tong University School of Medicine, Shanghai, China

**Keywords:** Stathmin, Mutant p53, Oncogene, Oral squamous cell carcinoma

## Abstract

**Background:**

The aim of this study was to investigate the oncogenic function and regulatory mechanism of stathmin in oral squamous cell carcinoma (OSCC).

**Methods:**

Two-dimensional electrophoresis and liquid chromatography-tandem mass chromatography were applied to screen differentiated proteins during carcinogenesis in OSCC. Cell Counting Kit-8 (CCK-8) assays, colony formation, migration, flow cytometry, immunofluorescence and a xenograft model were used to detect the function of stathmin. The correlation between stathmin and p53 expression was analyzed using immunohistochemistry. Mutant/wild type p53 plasmids and small interfering RNA were used to examine the regulation of stathmin. Chromatin immunoprecipitation assays and luciferase assays were performed to detect the transcriptional activation of stathmin by p53.

**Results:**

Overexpression of stathmin was screened and confirmed in OSCC patients and cell lines. Silencing expression of stathmin inhibited proliferation, colony formation and migration and promoted apoptosis. Poly ADP ribose polymerase (PARP) and cyclin-dependent kinase 1 (cdc2) were activated after silencing the expression of stathmin. Suppression of tumorigenicity was also confirmed in vivo. Mutant p53 transcriptionally activated the expression of stathmin in HN6 and HN13 cancer cells, but not in HN30 cells harboring wild type p53.

**Conclusions:**

These results suggest that stathmin acts as an oncogene and is transcriptionally regulated by mutant p53, but not by wild-type p53. Stathmin could be a potential anti-tumor therapeutic target in OSCC.

**Electronic supplementary material:**

The online version of this article (doi:10.1186/s13046-017-0575-4) contains supplementary material, which is available to authorized users.

## Background

Oral squamous cell carcinoma (OSCC) accounts for more than 300,000 new cases each year and unfortunately has poor clinical outcomes with a 5-year survival rate of only 50–60%, which is even lower in patients with locally advanced disease [1, 2]. Despite substantial progress made in the past few decades to understand the mechanism of carcinogenesis, treatment strategy has developed slowly for OSCC. In terms of treatment, surgery remains the best option for OSCC patients but it is not effective on late-stage metastatic tumors [3]. Betel quid, alcohol, tobacco and human papillomavirus are widely regarded as carcinogens for oral cancer. It is generally accepted that carcinogenesis of OSCC is a” Darwinian process” that involves a series of gene mutations causing the selected growth of mutated cells which replace normal cells in a specific region [4]. *TP53, CDKN2A, PIK3CA, PTEN, NOTCH* and *EGFR* are the most frequently mutated genes in head and neck cancer [5]. Mutations in the *TP53* gene have been widely studied in OSCC, with about 75% of mutations occurring in the DNA binding domain. Mutations in this region can activate a series of oncogenes to promote tumor progression, while wild-type p53 (wtp53) may inhibit tumor growth [6]. Neomorphic mutant p53 (mutp53) activities are spread over multiple levels, impinging on chromatin structure, transcriptional regulation and microRNA maturation, shaping the proteome and cellular metabolic pathways, and also exerting cytoplasmic functions and displaying cell-extrinsic effects [7]. Patients carrying mutant p53 are always resistant to chemotherapy and radiotherapy with poor prognosis [8]. Therefore, it is crucial to deeply investigate the molecular network involved in gain-of-function (GOF) mutations of p53 in OSCC.

Stathmin 1 (hereafter referred to as stathmin), also known as oncoprotein 18/op18, is a microtubule-destabilizing phosphoprotein that is ubiquitously expressed and involved in regulating the global dynamics of mitotic and interphase microtubules [9]. Stathmin prevents the incorporation of αβ-tubulin dimers in growing microtubules, resulting in eventual microtubule destabilization [10]. Stathmin overexpression has been reported in many types of human cancers and is associated with promotion of cancer cell proliferation, migration, invasion and resistance to paclitaxel [11–18]. In our previous study, we observed an interaction between stathmin and p53 in OSCC [19]. However, the relationship between stathmin and mutant/wild-type p53 remains unclear. Conflicting evidence has been reported regarding the relationship between stathmin and mutant/wild-type p53 expression [20, 21]; thus, the regulatory mechanism between mutp53 and stathmin in OSCC requires further investigation.

In this study, we demonstrate that stathmin was overexpressed in OSCC patients and cell lines, which promoted progression and tumorigenesis. Mechanistically, stathmin was regulated by mutp53 but not by wtp53 in OSCC.

## Methods

### Two-dimensional electrophoresis (2-DE) and liquid chromatography-tandem mass chromatography (LC-MS/MS)

2-DE and LC-MS/MS in human immortalized oral epithelial cells (HIOECs) and in HB96 cells were described thoroughly in our previous study [22]. Briefly, HIOECs and HB96 cells were lysed, sonicated and protein was quantified. First-dimensional IEF was completed with an IPGphor IEF System (Amersham Biosciences, Uppsala, Sweden) and second-dimensional SDS–PAGE was performed with a Hoefer SE 600 Ruby System (Amersham). Differentially expressed protein spots were excised and digested for mass spectroscopy. The peptide mixtures were isolated and identified by a Finnigan LTQ mass spectrometer coupled with the Surveyor HPLC system (Thermo, Sunnyvale, CA). Differentially expressed protein identification in MS/MS raw data was determined using the SEQUEST program in the BioWorks 3.1 software suite (University of Washington, licensed to Thermo Finnigan) based on the International Protein Index human database version 3.15.1.

### Immunohistochemistry

For immunohistochemistry, tissue samples from 60 patients and 16 healthy subjects were prepared according to the following protocol for immunohistochemical staining. Briefly, the sections were heated by water bath at 100 °C with citrate buffer solution (pH 6.0) for 20 min to retrieve antigen. The primary antibodies were rabbit monoclonal anti-stathmin (Abcam, ab52630, dilution 1:500; Cambridge, MA) and mouse monoclonal anti-human p53 (Abcam, ab28, 1:500). Microscopic examination of stathmin immunohistochemical staining was conducted by two blinded pathologists. The intensity of the stathmin immunoreaction was scored as following: 0 = negative, absence of stained cells; 1 = weak; 2 = moderate; 3 = strong. The immunohistochemical staining score was calculated by multiplying the percentage of positive cells and the staining intensity as described in the literature [23]. This study was approved by the Ethics Committee of Ninth People’s Hospital, Shanghai Jiao Tong University School of Medicine. Informed consent was obtained from all patients for use of their tissues.

For immunofluorescence, Alexa Fluor 488-Affini goat anti-rabbit IgG (Abcam, ab150077, 1:250) was used as the secondary antibody. DAPI was used to stain cell nuclei. The morphology of microtubules was observed using rat monoclonal anti-tubulin antibody (Abcam, ab6160, 1:500) under a laser scanning confocal microscope (LSM-710, Carl Zeiss, Gottingen, Germany).

### Cell culture

The cell lines used in the present study were HIOEC, HB96 (derived cancerous cell line from HIOEC), HN4, HN6, HN12, HN13, CAL27, HN30 and SCC25. HIOECs and HB96 cells were obtained from our previously established in vitro cellular carcinogenesis model of OSCC [24]. HN4, HN6, HN12 and HN13 lines were obtained from tongue squamous cell carcinoma patients, while HN30 cells were derived from a pharyngeal carcinoma. All of the HN lines were provided by the University of Maryland Dental School, Baltimore, MD. CAL27 and SCC25 lines were purchased from ATCC (Manassas, VA). The mutant phenotype of *TP53* in cells was detected as described by Song et al. [25]. All OSCC cell lines used in this study were HPV negative. HIOECs were cultured in defined keratinocyte-SFM (Gibco, Carlsbad, CA) and SCC25 were cultured in DMEM/F12 (Gibco). The other cell lines were cultured in DMEM (Gibco). Both DMEM and DMEM/F12 were supplemented with 10% fetal bovine serum, 1% glutamine, and 1% penicillin-streptomycin (Gibco). All cells were cultured in a humidified atmosphere of 5% CO_2_ at 37 °C.

### Western blot assay

Protein extraction and western blots were performed as previously described [26]. Membranes were probed with primary rabbit antibodies against stathmin, p53, beta-actin, cyclin D1, bcl-2 and GAPDH, purchased from Abcam; or with antibodies against cleaved-PARP, PARP, cyclin B1, cdc2 and p-cdc2 (Tyr15), purchased from Cell Signaling Technology, Danvers, MA; and finally with fluorescent-based anti-rabbit IgG secondary antibody (Fermentas, Vilnius, Lithuania). Immunoreactive bands were scanned and analyzed using Image J software (NIH, Bethesda, Maryland) and the Odyssey Infrared Imaging System (LI-COR Biosciences, Lincoln, NE).

### Real-time PCR assay

Total RNA from cultured cells was isolated at 80% confluence with TRIzol reagent (Takara, Dalian, China) according to the manufacturer’s instructions. Total RNA (1 μg) was reverse transcribed into first strand cDNA with PrimeScript reverse transcriptase (Takara, Dalian, China), random primers and oligo dT primer in a 20 μl reaction mixture according to the manufacturer’s protocol. Real-time PCR was performed with the StepOnePlus™ Real Time System (Applied Biosystems, Foster City, CA). The primer sequences were as follows. Stathmin: 5′-CTCGGACTGAGCAGGACTTTC-3′ (forward) and 5′-ATTCTTTTGACCGA GGGCTG-3′ (reverse); p53: 5′-CCAGAAAACCTACCAGGGCA-3′ (forward) and 5′-GAATGCAAGAAGCCCAGACG-3′ (reverse); and GAPDH: 5′-CCTCTGACTTCAACAGCGAC-3′ (forward) and 5′-TCCTCTTGTGCTCTTGCTGG-3′ (reverse). Relative quantification of mRNA levels compared to the internal control gene GAPDH was calculated according to the 2^-ΔΔct^ method. Melting and amplification curves were performed to determine the specificity of stathmin amplification. All samples were assayed in triplicate.

### Small interfering RNA (siRNA) and plasmid transfection

The siRNAs against stathmin, p53 and scrambled control sequence were designed as follows. siRNA-stathmin: 5′-CAGCCCUCGGUCAAAAGAAtt-3′ (si#1); 5′-GCUGCCAAACUGGAACGUUtt-3′ (si#2); siRNA-p53: 5′-GTCCAGATGAAGCTCCCAGAAdTdT-3′; siRNA–negative control (NC): 5′-UUCUCCGAACGUGUCACGUdTdT-3′. siRNAs were chemically synthesized by Shanghai GenePharma Co., Shanghai, China. In addition, si#2 sequence was packaged into the lentiviral pGLV-h1-GFP-puro vector for in vivo experiments. Cells were transfected using Lipofectamine™ 3000 Transfection Reagent (Invitrogen, Carlsbad, CA) according to the manufacturer’s instruction. Transfection efficiency for the siRNAs in HN4 and HN13 cells was 50% ~ 70%. Transfected cells (1000–5000 per well) were seeded in 96-well plates and cultured for 5 days. Cell Counting Kit-8 (CCK-8; Dojindo, Kumamoto, Japan) solution (10 μl) was added to each well followed by incubation at 37 °C for an additional 3 h. The absorbance at 450 nm was measured to assess cell viability. The experiment was performed in triplicate.

### Colony formation assay

After transient transfection for 24 h with siRNA, 1000 cells were plated in 6-well plates for 3 weeks until macroscopic colonies were visible. The colonies were fixed and stained using crystal violet. Colonies of more than 50 cells were counted under a dissecting microscope. The data were expressed as means ± SD from three independent experiments.

### Transwell assay

Briefly, 3–5 × 10^4^ transfected cells were seeded in the upper chambers of filter inserts containing serum-free medium. The lower chambers held 500 μl medium per well containing 10% FBS in 24-well plates. After 24–36 h, the cells were stained using 5% crystal violet. The number of cells migrating to the lower chamber was counted. The experiment was performed in triplicate.

### In vitro wound healing assay

Transfected cells were incubated overnight yielding confluent monolayers suitable for wounding. Wounds were scratched using a 10 μl pipette tip. At 0 h and 24 h, photographs were taken with a Nikon DMCI microscope (Nikon, Tokyo, Japan). The distance migrated by invasive cells to heal the wound area during this period was measured.

### Apoptosis assay

Cells transiently transfected with siRNA were harvested 48 h after incubation. These cells were then quantified by flow cytometry using the FITC-Annexin V Apoptosis Detection Kit (BD Biosciences, Franklin Lakes, NJ) according to the manufacturer’s instructions. The experiments were performed in triplicate.

### Tumorigenicity assay in vivo

To evaluate the antitumor effect of stathmin in vivo, an OSCC xenograft model was generated in BALB/C nude mice (4–6 weeks old, 18–22 g) by subcutaneously injecting 2 × 10^6^ HN13 cells after lentivirus transfection. The transfected cells were selected in medium containing 4 μg/μl puromycin to harvest stable stathmin-knockdown cells and scrambled control cells. Tumors were measured once per week for 5 weeks, and tumor weights were measured after sacrifice.

### Plasmid construction

To construct the stathmin expression vector, the code domain sequence (CDS; NM_203401.1) was cloned into the eukaryotic expression vector pcDNA3.1 (Invitrogen). Wild-type p53 CDS (NM_ 000546) and a mutant sequence template (Shanghai GenePharma; 524A > G, 733 U > G, 844 U > C) were cloned into pcDNA3.1.

### Chromatin immunoprecipitation assay (ChIP)

To explore the regulatory mechanism of stathmin in OSCC, ChIP was conducted in OSCC cell lines. ChIP assays were performed according to the manufacturer’s instructions using the SimpleChIP Enzymatic Chromatin IP Kit (magnetic beads; CST). Briefly, p53 and protein complexes were cross-linked inside HN6, HN13 and HN30 cells by the addition of formaldehyde (1% final concentration) to the cells in culture. Chromatin was digested with micrococcal nuclease and sonicated into 150–900 bp DNA/protein fragments. An aliquot of the cross-linked protein complexes was immunoprecipitated by incubation with either p53-specific antibody (CST) or IgG antibody overnight at 4 °C with rotation. Chromatin-antibody complexes were isolated from solution by incubation with ChIP-Grade Protein G Magnetic Beads for 1 h at 4 °C with rotation. The bead-bound immune complexes were then washed and eluted from the beads with elution buffer. Eluates were heated at 65 °C overnight to reverse the formaldehyde cross-linking and DNA was extracted. DNA samples from chromatin immunoprecipitation preparations were analyzed by PCR using primers spanning the stathmin gene (NM_203401.1) in the promoter region (forward, 5′-AATGGGGAGCTGGTTCGGA-3′; reverse, 5′-GTGTAGTCCTGTCCCGGAGG-3′). The transcription factor binding sites of p53 were predicted using the website of JASPAR (http://jaspar.genereg.net/) [27] and PROMO [28, 29]. The two predicted binding sites of CCAGGCCCACACCTG and GCAACCCCCGGCATT were amplified using the above promoter primers. Primers used in the real-time PCR mentioned above amplified the CDS region. The nonpromoter regions were used as control.

### Luciferase assay

Luciferase assays were used to confirm the transcriptional activation leading to p53 GOF. 293 T and SCC25 cells were transiently transfected using transfection reagent Lipofectamine 3000 (Invitrogen). Cells were seeded in 12-well plates (1 × 10^5^ cells/well) and grown to 70–80% confluence. Each stathmin promoter-luciferase construct was co-transfected into cells with pRL-TK (TK promoter Renilla luciferase construct as internal control). Luciferase activity was determined 24–48 h after transfection using a dual luciferase reporter assay system (Beyotime, Shanghai, China). Cell lysates (200 μl/well) were used for measurement of relative luciferase units in a luminometer by first mixing 20 μl of cell lysates with 100 μl of luciferase assay reagent to measure firefly luciferase activity, then subsequently adding 100 μl of Renilla luciferase reagent to measure Renilla luciferase activity. Data were normalized to Renilla luciferase activity (internal control) and expressed as arbitrary units. siRNA against p53 was co-transfected with p53 plasmids. NSC319726 is a mutp53 (R175H) reactivator, exhibiting growth inhibition in cells expressing mutp53 and showing no inhibition of wtp53 cells [30]. NSC319726 was added after transfection with the mutp53 plasmids. All experiments were performed in triplicate.

### Statistical analysis

All data were analyzed using the statistical software SPSS 13.0 for Windows (SPSS Inc., Chicago, IL). Student’s *t*-test and one-way analysis of variance were used to analyze immunohistochemical results. All hypothesis-generating tests were two-tail at a significance level of 0.05 (*P < 0.05, **P < 0.01). Data were presented as means ± standard error of the mean.

## Results

### Identification of stathmin overexpression in OSCC

**Fig. 1:**
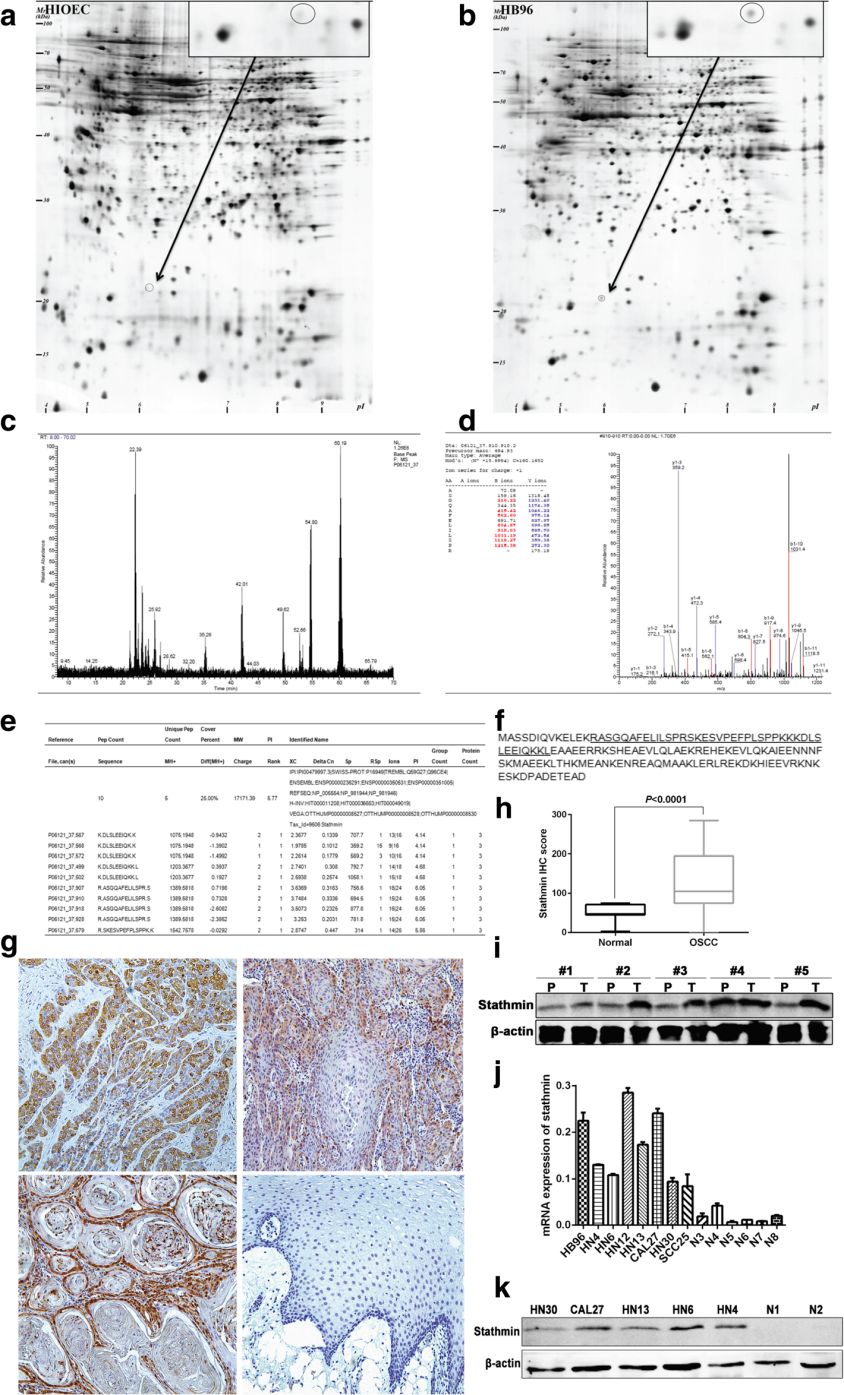
Screen and identification of stathmin expression in OSCC cell lines and tissue samples. Using 2-DE and silver staining, comparing to the human immortalized oral epithelial cells (**a**), overexpressed protein spot (Molecular Weight = 17,171.39 Da, *pI =* 5.77) was found in the derived cancerous cell line of HB96 (**b**). The peptide fingerprint of the differentially expressed spot was identified by MALDI-TOF MS/MS (**c**, **d**). Protein identification with the MS/MS raw data was performed as stathmin using the SEQUEST program in the BioWorks 3.1 software suite (University of Washington, licensed to Thermo Finnigan) based on the IPI Human database version 3.15.1 (**e**). The reference amino acids sequence of stathmin was shown with underlined characters (**f**). Representative images show immunohistochemical staining against stathmin in poorly, moderately, and well differentiated OSCC, and normal oral mucosa tissue. Original magnification: ×200 (**g**). The stathmin IHC score in OSCC patients was higher than normal controls (**h**). Stathmin expression was detected in tumor (T) and paracancerous (P) tissues from five OSCC patients (P) using western blot (**i**). Overexpression of stathmin at the mRNA and protein levels was confirmed in OSCC lines compared to the normal oral mucosa (**j**, **k**)

Two types of cells from in vitro cellular carcinogenesis, HIOEC and derived cancerous cells (HB96) were used for 2-DE analysis **(**Fig. 1a, b **)**. Fifty-four differentially expressed protein spots were identified in our previous study, and LC MS/MS was used to generate a chromatogram, depicting protein digests from differentially expressed spots [31]. Stathmin was overexpressed during carcinogenesis of immortalized oral epithelial cells to squamous carcinoma cells **(**Fig. 1c–f **)**. To verify the results of our proteomics analysis, stathmin expression was measured in OSCC tissues and cell lines. There was a significant correlation between stathmin immunohistochemical scores and pathological differentiation grades (*P* < 0.001), with a poorer pathological differentiation grade indicating a higher staining score **(**Table 1 **,** Fig. 1g **)**. No significant correlation was found between stathmin expression and other clinicopathological parameters. The stathmin immunohistochemical score in OSCC was significantly higher than in normal oral mucosa (141.3 ± 10.3 vs 52.4 ± 4.7, *P* < 0.001**;** Fig. 1h **)**. Moreover, stathmin expression was higher in tumor tissues (T) than in precancerous tissues (P) in 4 out of 5 OSCC patients **(**Fig. 1i **)**. Real-time PCR showed higher stathmin mRNA levels in 8 OSCC lines compared to 6 normal oral mucosal cell lines **(**Fig. 1j **)**. Western blot analysis also showed stathmin overexpression in OSCC lines compared to controls **(**Fig. 1k **)**. To summarize, overexpression of stathmin was screened and confirmed in OSCC.

**Table 1:** The correlation between stathmin expression and clinical characteristics in OSCC

Characteristics	Patients (%)	Stathmin score		parametric test value	*P* value
		Mean	SEM		
Gender					
Male	35(58.3)	151.6	14.1	*t* = 1.189	0.239
Female	25(41.7)	126.8	14.7		
Age					
< 60 years	34(56.7)	143.2	12.4	*t* = 0.218	0.828
≥ 60 years	26(43.3)	138.7	17.7		
T stage					
T1	17(28.3)	145.6	21.5	*F* = 0.039	0.990
T2	19(31.7)	142.6	19.7		
T3	8(13.3)	136.3	29.8		
T4	16(26.7)	137.5	16.8		
N stage					
cN0	41(68.3)	137.9	12.0	*t* = −0.470	0.640
cN+	19(31.7)	148.4	20.0		
TNM stage					
I/II	28(46.7)	132.7	14.7	*t* = −0.775	0.441
III/IV	32(53.3)	148.8	14.5		
Pathologic differentiation					
Well	30(50.0)	100.8	10.3	*F* = 14.757	***< 0.001*** ^*^
Moderately	22(36.7)	162.5	17.2		
poorly	8(13.3)	234.4	18.6		
Smoking status					
Yes	21(35.0)	143.8	15.6	*t = 0.181*	0.857
No	39(65.0)	139.9	13.6		
Alcohol use					
Yes	15(25.0)	128.7	19.3	*t* = −0.702	0.486
No	45(75.0)	145.4	12.2		

^*^Stathmin immunohistochemical scores correlated significantly with pathological differentiation grades, a poorer pathological differentiation grade indicated a higher staining score

### Stathmin overexpression promotes progress of OSCC in vitro and in vivo

**Fig. 2:**
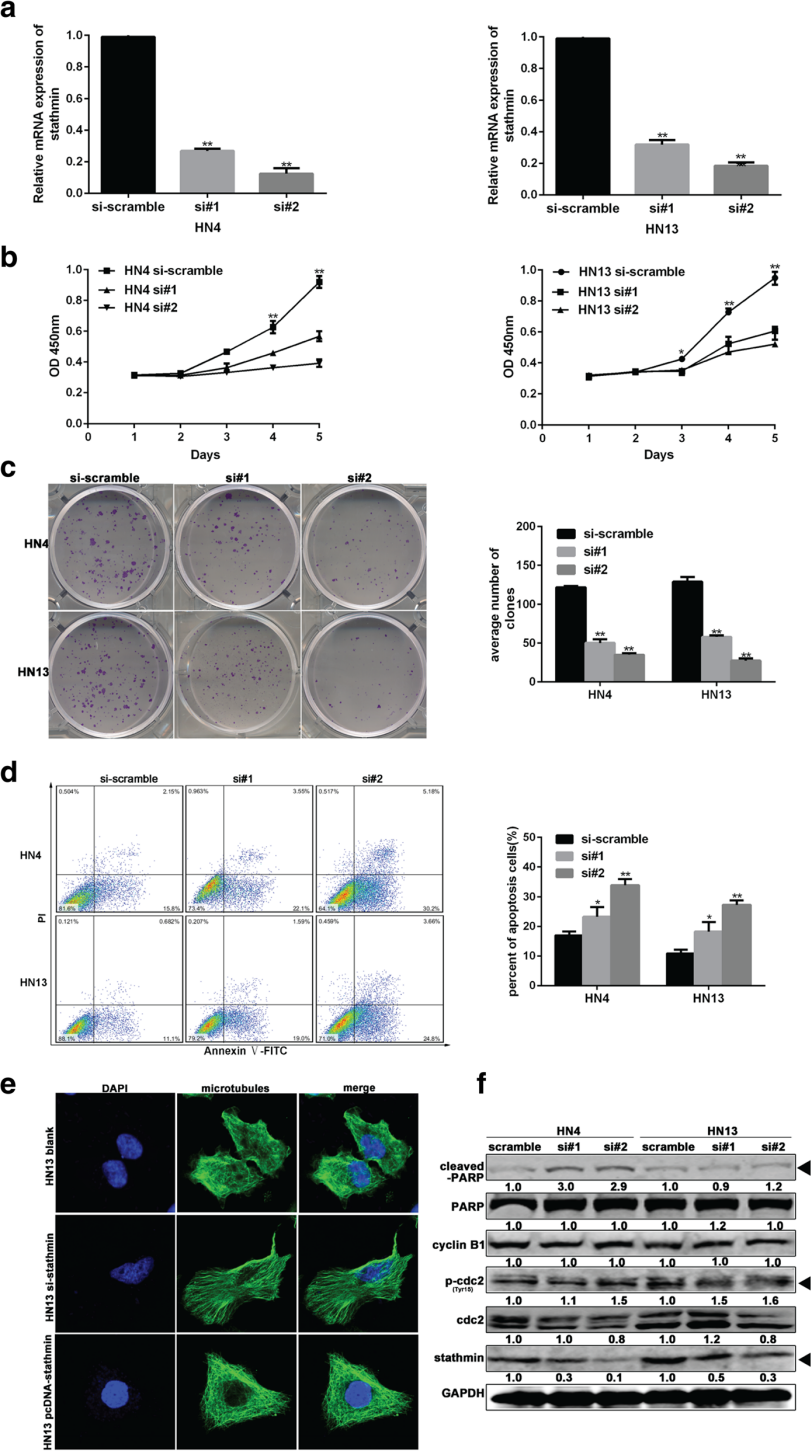
Down-regulation of stathmin expression inhibited the progress of OSCC in vitro. The mRNA of stathmin was detected after transfection with siRNA (#1, #2) against stathmin for 24 h in HN4 and HN13 (**a**). Cellular growth was inhibited after transfection with siRNA-stathmin in the HN4 and HN13 cells using a CCK-8 kit (**b**), colony formation was also inhibited compared to the scramble siRNA (**c**). The proportion of apoptotic cells increased markedly (*lower* and *upper right* fraction) in the HN4 and HN13 cells transfected with siRNA-stathmin for 48 h compared to the control using the PI and Annexin V staining (**d**). Morphological alterations of microtubules were stained by α-tubulin (*green*) and nuclei were stained by DAPI (*blue*) in HN13 cells transfected with siRNA-stathmin and pcDNA-stathmin plasmids (**e**). The downstream signaling molecules were detected by western blot after siRNA transfection for 48 h (**f**). **: *P* < 0.01, *: *P* < 0.05

**Fig. 3:**
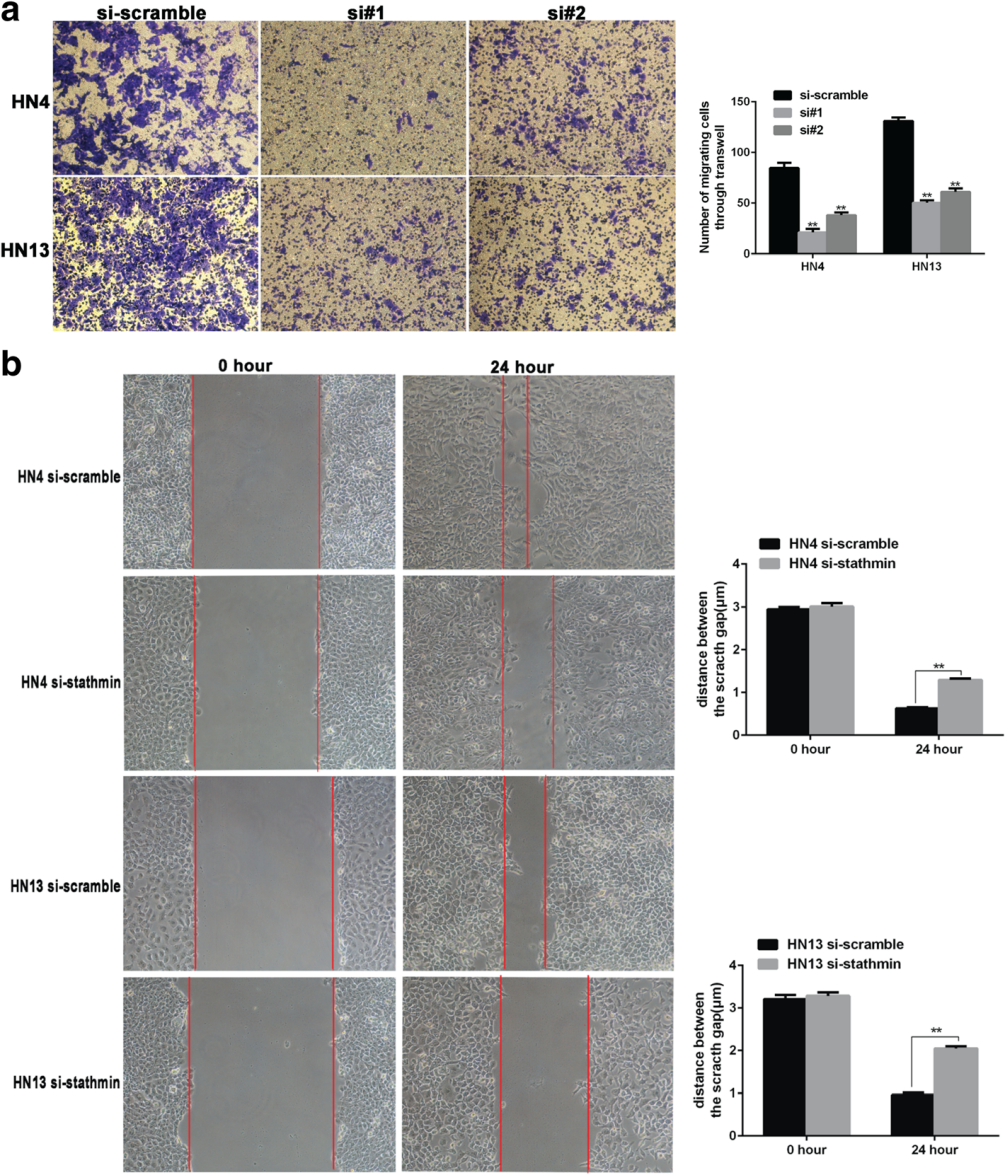
The migration capacity was inhibited in HN4 and HN13 after silence stathmin. Using a transwell assay, 3–5 × 10^4^ cells after transfection for 24 h were seed in the upper chamber with serum free medium, and the lower chamber was DMEM medium containing 10% FBS. After incubation for 36 h, the number of migrating cells on the membrane were stained with crystal violet and counted under microscope. **a** Using a wound healing assay, HN4 and HN13 cells transfected with siRNA-stathmin (#2) were seed in 6-well plate with a confluence of 90%. The distance migrated by the invasive cells to heal the wound area during this period was measured (**b**). **: *P* < 0.01

To explore the biological function of stathmin in OSCC, we firstly knocked down the expression of stathmin by transient transfection of siRNA-stathmin (si#1 and si#2) in HN4 and HN13 cell lines. After transfection, stathmin mRNA expression was greatly reduced compared to scrambled controls (Fig. 2a), accompanied by a significant decrease in cell proliferation in both HN4 and HN13 lines **(**Fig. 2b **)**. Likewise, colony formation was significantly inhibited in both HN4 and HN13 lines **(**Fig. 2c **)**. The mean percentage of apoptotic cells in transfected HN4 and HN13 lines was 25.7% (si#1) and 35.4% (si#2) in HN4 cells, and 20.6% (si#1) and 28.5% (si#2) in HN13 cells, which was significantly higher than the percentage in control cells (17.95% and 11.78%; *P* < 0.05**;** Fig. 2d **)**. Given the important role of stathmin in microtubule destabilization, we sought to observe morphological changes regulated by stathmin. After knockdown of stathmin expression, microtubules became longer and well arranged in a comb-like appearance (middle panel, Fig. 2e). However, after ectopic expression of stathmin, the microtubules reverted into shorter, chaotic nest-like figures (lower panel), in contrast to the long hair-like microtubules observed in control HN13 cells (upper panel). Using western blot analysis, elevated levels of cleaved-PARP indicated a pro-apoptotic effect after knockdown of stathmin in HN4 and HN13 lines. As a critical G2/M phase regulator protein, upregulated phosphorylation of cdc2 after knockdown of stathmin inhibited mitosis **(**Fig. 2f **)**. Moreover, the anti-apoptotic effect of stathmin overexpression in OSCC correlated with upregulation of bcl-2 and cyclin D1 expression **(**Additional file 1: Fig. S1). Furthermore, migration capacity was significantly suppressed as indicated in transwell **(**Fig. 3a **)** and wound-healing assays **(**Fig. 3b **)** in HN4 and HN13 cells after silencing of stathmin. Taken together, proliferation and migration capacity were inhibited after knockdown of stathmin in OSCC.

**Fig. 4:**
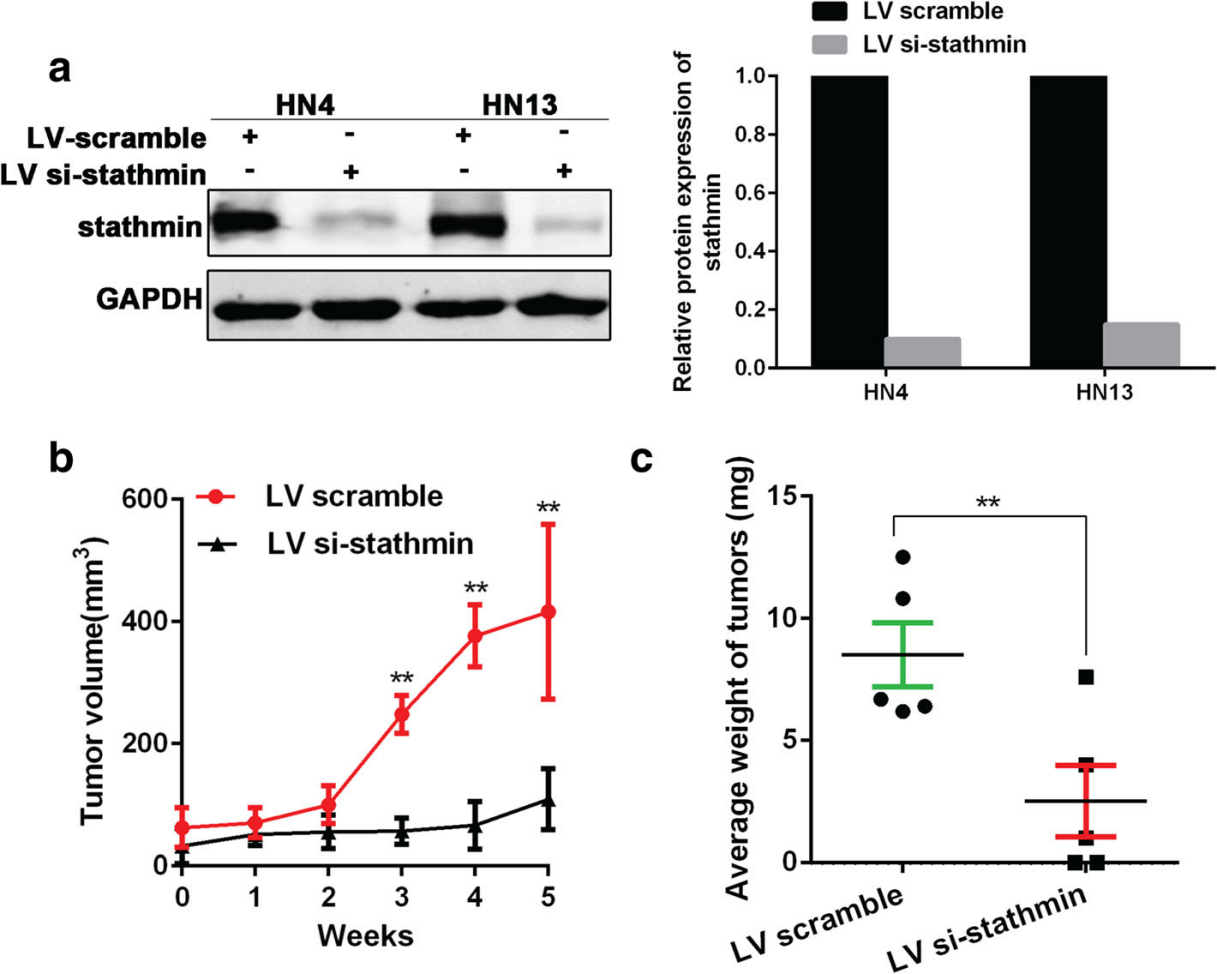
Tumor growth was suppressed after lentivirus transduction in HN13 cells in nude mice. Lentiviral stable cell lines of stathmin silence was constructed and confirmed in HN4 and HN13 cells. The relative protein expression of stathmin was analyzed using Image J software (**a**)**.** Using a subcutaneous tumorigenesis model in nude mice, xenograft tumors grew slower in mice injected with HN13 cells transfected with siRNA-stathmin than those transfected with scramble (**b**). The tumor weight in knockdown group was smaller than scramble control (**c**). **: *P* < 0.01, *: *P* < 0.05

To confirm the in vitro results, lentivirus-infected stable cell lines in which stathmin was silenced were constructed and verified using HN4 and HN13 cells **(**Fig. 4a **)**. The xenograft tumor model was established with HN13 cells transfected by lentivirus vector. Tumors in the si-stathmin group grew much more slowly compared to the scrambled group **(**Fig. 4b **)**. Likewise, tumor weight was significantly smaller than in the scrambled group **(**Fig. 4c **)**. In summary, overexpression of stathmin promoted tumor growth in vitro and in vivo in OSCC.

### Positive correlation between stathmin and p53 expression in OSCC tissues

**Fig. 5:**
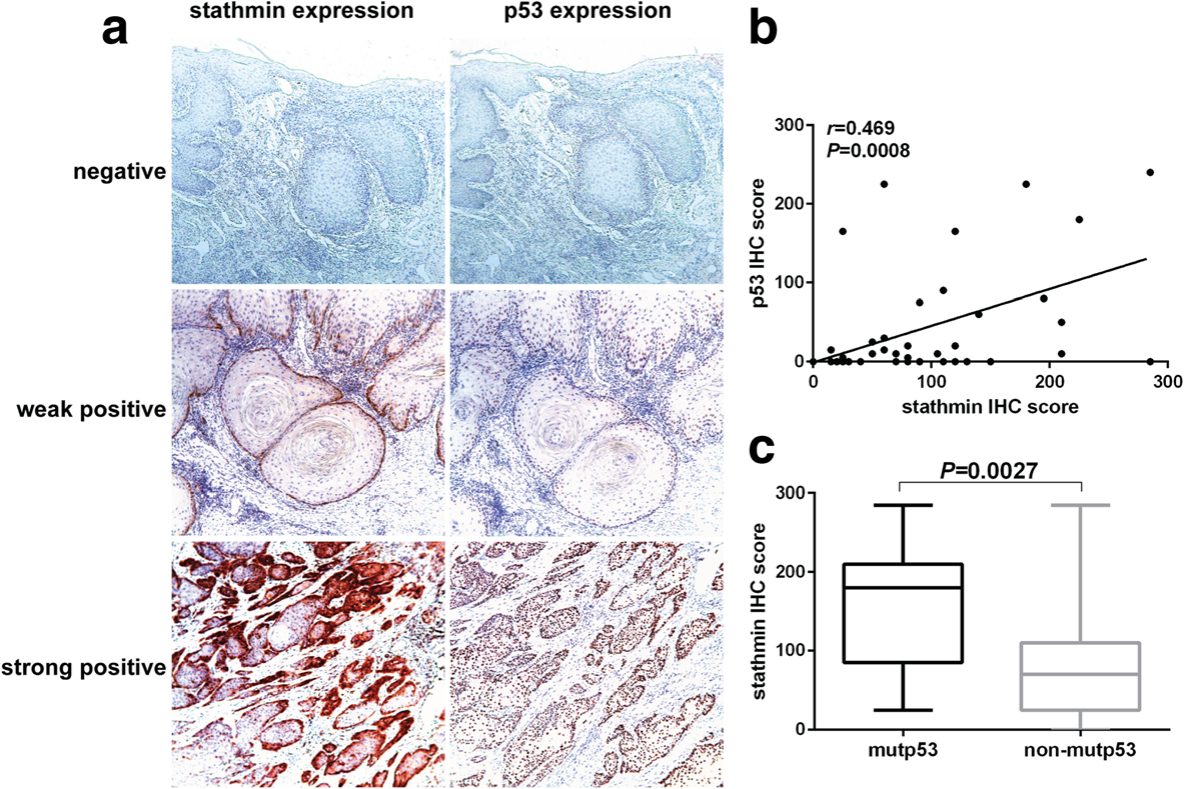
Stathmin overexpression correlated with mutant p53 expression in OSCC patients. Stathmin and p53 expression was detected in 48 OSCC patients using immunohistochemistry (**a**). The positive correlation between stathmin and p53 expression was demonstrated in a scatter plot (*r* = 0.469, *P* = 0.0008) (**b**). The stathmin IHC score was higher in patients with mutp53 than those with wtp53 (*P* = 0.0027) (**c**). Original magnification: ×200, **: *P* < 0.01, *: *P* < 0.05

To explore the relationship between stathmin and p53 expression, immunohistochemical staining of stathmin and p53 was performed in 48 OSCC patients with paired slides for both proteins, and a positive correlation was found between stathmin and p53 expression (*r* = 0.469, *P* = 0.008**;** Fig. 5a, b **)**. In general, normal levels of wtp53 protein expression are too low to detect by routine immunohistochemistry [32], but a more robustly positive percentage of nuclear p53 staining (>30%) in tumor cells may indicate the presence of mutp53 protein [33, 34]. Accordingly, the immunohistochemical score of stathmin expression was significantly higher in patients with mutp53 expression than in those with wtp53 expression (153.3 ± 27.3 vs 74.9 ± 10.1, *P* = 0.003**;** Fig. 5c **)**, indicating a potential positive correlation between mutp53 and stathmin expression.

### Stathmin expression is regulated by mutp53, not wtp53 in OSCC

**Fig. 6:**
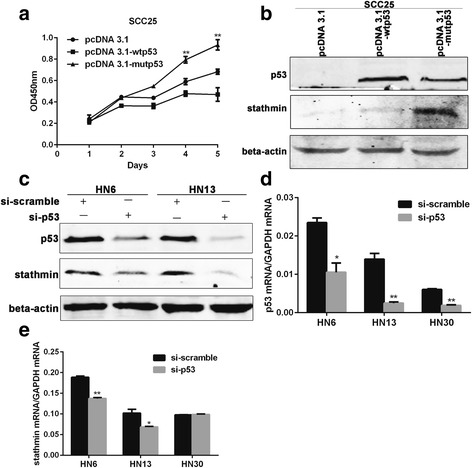
Mutant p53 promoted the expression of stathmin. After wtp53, mutp53 (R175H, G245C and R282W) plasmids and vectors were transfected into SCC25 cells, cells transfected with mutp53 grew more aggressively than those with wtp53 (**a**). In SCC25 cells transfected with pcDNA3.1 vector, pcDNA3.1-wtp53 and pcDNA3.1-mutp53 for 48 h, upregulation of stathmin was found in cells transfected with mutp53 but not wtp53 (**b**). Decreased stathmin expression was found accompanied by p53 knockdown for 48 h in two mutant p53 cell lines, HN6 (H179L) and HN13 (V173 L) (**c**). Decreased stathmin mRNA levels were found accompanied by knockdown of p53 for 48 h in HN6 and HN13 cells but not wtp53 HN30 cells (**d**, **e**). **: *P* < 0.01, *: *P* < 0.05

For this study, we used the SCC25 cell line, which has undetectable levels of p53 protein resulting from the deletion of two base pairs in codon 209 [35, 36], and also examined three p53 mutants (R175H, G245C and R282W) which were found to promote invasive cell growth in head and neck squamous cell carcinoma [37]. When wtp53 and mutp53 (R175H, G245C and R282W) plasmids and vectors were transfected into SCC25 cells, the cells transfected with mutp53 grew more aggressively than those transfected with wtp53 **(**Fig. 6a **)**, but significant upregulation of stathmin expression was only observed in cells transfected with mutp53 **(**Fig. 6b **)**. When siRNA against p53 was transfected into the two mutp53 cell lines, HN6 (H179L) and HN13 (V173 L), we found decreased stathmin expression accompanied by p53 knockdown at both the protein and mRNA levels **(**Fig. 6c, e **)**. However, after transfection with siRNA against p53 in HN30 (wtp53), the transcription of stathmin did not change in relationship to p53 mRNA levels **(**Fig. 6d, e **)**, indicating that stathmin expression was regulated by mutp53 but not wtp53 in OSCC.

**Fig. 7:**
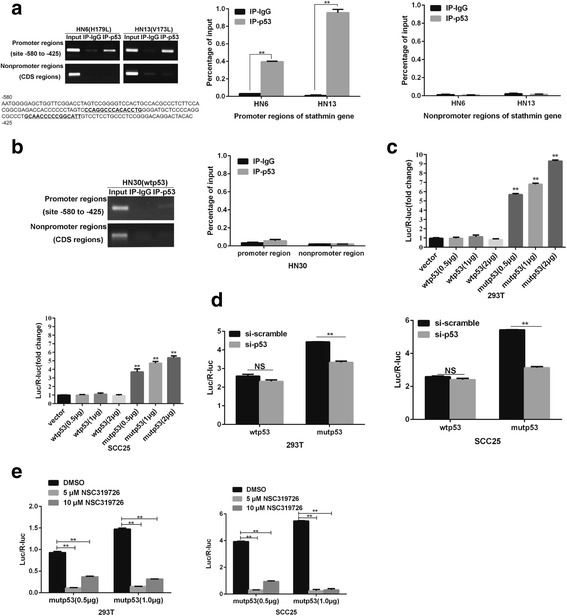
Mutp53 specifically bound to and regulated the stathmin promoter activity. The binding of p53 to the stathmin promoter (two predicted binding sites were amplified using the promoter region primers spanning from −580 to −425, upstream) was detected by a chromatin immunoprecipitation assay in HN6 (mutp53 H179L) and HN13 (V173 L) cells. The p53 consensus binding sites were indicated in bold (**a**). There was no binding between p53 and the stathmin promoter in HN30 cells with wtp53 (**b**). The promoter activity was examined in the absence and presence of the p53 expression vector, and increased promoter activity in a dose-dependent manner was found in 293 T and SCC25 cells transfected with mutp53 compared to those with vector and wtp53 (**c**).The promoter activity of stathmin was detected after transfection with siRNA against p53 for 24 h in mutp53-transfected 293 T and SCC25 cells; however, no change of promoter activity was found in cells transfected with wtp53 (**d**). The promoter activity of stathmin was detected in 293 T and SCC25 cells co-transfected with mutp53 (0.5 μg and 1.0 μg) and stathmin promoter luciferase plasmids for 24 h, and then treated with NSC319726 for 24 h (0 μM, 5 μM, 10 μM) **(e)**. **: *P* < 0.01, *: *P* < 0.05

We hypothesized that direct transcriptional regulation was responsible for regulating stathmin expression by mutp53. A publically available schematic diagram of the binding motif of p53 **(**Additional file 2: Fig. S2) was evaluated to identify two binding sites found in the mutp53 lines of HN6 and HN13. Upon ChIP-PCR analysis, mutp53 bound to the promoter region of stathmin but not to the CDS **(**Fig. 7a **)**, while in the wtp53 line of HN30, p53 did not bind at all to the promoter region of stathmin **(**Fig. 7b **)**, suggesting that mutp53 but not wtp53 bound the stathmin promoter region. To test whether mutp53 could transcriptionally activate stathmin expression, a dual-luciferase reporter system was developed to examine promoter activity. The promoter activity of stathmin significantly increased in a dose-dependent manner after transfection with mutp53 compared to the control vector and wtp53 **(**Fig. 7c **)**. After co-transfection with siRNA against p53 and p53 plasmids, the promoter activity of stathmin significantly decreased in cells transfected with mutp53 **(**Fig. 7d **)**. Previously, the reactivator of mutp53, NSC319726, exhibited growth inhibition in cells with mutp53 (R175) and showed no inhibition in cells with wtp53 [30]. In our study, deceased promoter activity of stathmin was found in mutp53-transfected 293 T and SCC25 cells when treated with NSC319726 **(**Fig. 7e **)**. These results indicated that stathmin overexpression was transcriptionally activated by mutp53 but not by wtp53 in OSCC.

## Discussion

In this study, we demonstrated that overexpression of stathmin promoted tumor progression and was regulated by mutp53 in OSCC. Overexpression of stathmin has been detected in several malignancies. Stathmin overexpression in hepatoma promoted local invasion, polyploidy formation, early recurrence and poor prognosis, suggesting that stathmin can be an effective therapeutic target [11]. Proteins from pooled microdissected nasopharyngeal carcinoma and adjacent non-tumorigenic nasopharyngeal epithelial tissues were separated by 2-DE to find significant overexpression of stathmin in the tumors [17]. Stathmin knockdown by siRNA in melanoma cells drastically repressed cell proliferation and migration, whereas ectopic expression of stathmin increased cell proliferation and migration [18], consistent with our results. From the reports in various carcinomas, overexpression of stathmin was very consistent in several tumors and acted as a potential oncogene.

Recently, possible mechanisms that may underlie the multiple functions of stathmin in cancer have attracted much attention. In our study, cleaved PARP (the 89-kDa cleaved product) was increased after silencing stathmin, which indicated the pro-apoptotic effect of stathmin knockdown in OSCC. Furthermore, bcl-2 and cyclin D1 increased after ectopic expression of stathmin. In agreement with these results, silencing of stathmin resulted in decreased expression of bcl-2 and survivin proteins and activation of caspase-3 and caspase-9 [38, 39].

Cdc2, also known as cyclin-dependent kinase 1, mainly regulates the progression of the G2/M phase. A conserved tyrosine (Tyr15 in humans) leads to inhibition of cdc2; its phosphorylation is thought to alter ATP orientation, preventing efficient kinase activity. Activation of cdc2 (try15) blocked cells at the G2/M phase which then induced apoptosis. In contrast, cyclin B1, the partner of cdc2, was unaltered in our study. It has also been reported that expression of p53 and p21 increased after silencing stathmin in gallbladder carcinoma [40]. In our study, we confirmed that upregulation of p-cdc2 (try15) after stathmin silencing arrested the cell cycle and induced apoptosis in OSCC. The results suggest that the downstream signaling molecules of stathmin differ in various cancers.

Overexpression of stathmin was transcriptionally activated by mutp53 but not by wtp53 in OSCC in our study. In over 70% of cases, the TP53 mutations are missense, most frequently within the region of DNA binding. Although the spectrum of the TP53 missense mutations is vast – counting about 1800 different amino-acid changes– several hotspots in p53 mutants, particularly affecting residues R273, R248, R175 and G245, have been reported to be present with a higher frequency in head and neck cancer [41]. Cells expressing mutp53 exhibited aggressive cancer phenotypes, such as enhanced cell survival, proliferation, invasion and adhesion, altered mammary tissue architecture and invasive cell structures [42]. This was confirmed in SCC25 cells after transduction with mutp53 plasmids. Stathmin depletion caused a large percentage of the apoptosis occurring in both normal and cancer cell lines lacking p53 [43], while significant inhibition of proliferation was also observed in OSCC cell lines with mutp53. This suggests that stathmin depletion could be used therapeutically to induce apoptosis in tumors with or without p53 expression. In our study, three p53 mutants (R175H, G245C and R282W) were constructed into one plasmid to mimic the complicated mutations of p53 in OSCC. These three mutants contributed to the mutp53 gain of oncogenic function to promote invasive growth of head and neck cancer cells via inhibition of AMPK activation [37]. Upregulation of stathmin was also shown to be mediated by GOF mutations in p53 in human hepatoma [21], as well as in breast cancer cell lines harboring mutp53 [39]. The results of our study are in accord with the above reports. In type II high grade epithelial ovarian carcinomas, stathmin favored the binding and the phosphorylation of mutp53 by DNA-PKCS, eventually modulating mutp53 stability and transcriptional activity [44]. It was speculated that a positive feedback loop between mutp53 and stathmin synergistically promoted the progression of malignant tumors. However, wtp53 has also been reported to downregulate stathmin expression [20, 45]. This discrepancy may be related to separate molecular driving mechanisms in different tumors, but our results nonetheless provide new insight into the interaction between mutp53 and stathmin overexpression in OSCC, which extends the network driving mutp53. The transcriptional regulation of STMN1 by p53 varies in different carcinomas, which deepens our understanding of GOF p53-driven tumors. Our results confirm that stathmin is a novel target of mutp53 and jointly promotes tumorigenesis and tumor progression.

## Conclusions

In summary, we demonstrated that stathmin was overexpressed in OSCC, which promoted progression and tumorigenesis. Silencing stathmin activated cdc2 at try15 and cleaved PARP to induce cell cycle arrest and apoptosis in OSCC. Importantly, overexpression of stathmin was transcriptionally activated by GOF mutations of p53, not by wtp53. Thus, our findings indicate that stathmin may be a potential therapeutic target for the treatment of OSCC.

## Additional files

Additional file 1: Fig. S1. In HN13 and HN30 cells transfected with stathmin overexpression vector, increased stathmin, cyclin D1, and bcl-2 expression were found when compared to those transfected with pcDNA, while no change in p53 expression. (TIFF 139 kb)

Additional file 2: Fig. S2. The binding motif of p53 was predicted, utilizing data publically available on JASPAR (http://jaspar.genereg.net/). (JPEG 30 kb)

## References

[1] Torre LA, Bray F, Siegel RL, Ferlay J, Lortet-Tieulent J, Jemal A (2015). Global cancer statistics, 2012. CA Cancer J Clin.

[2] Leemans CR, Braakhuis BJ, Brakenhoff RH (2011). The molecular biology of head and neck cancer. Nat Rev Cancer.

[3] Malik UU, Zarina S, Pennington SR (2016). Oral squamous cell carcinoma: key clinical questions, biomarker discovery, and the role of proteomics. Arch Oral Biol.

[4] Feller L, Wood NH, Khammissa RA, Lemmer J (2010). Human papillomavirus-mediated carcinogenesis and HPV-associated oral and oropharyngeal squamous cell carcinoma. Part 1: human papillomavirus-mediated carcinogenesis. Head Face Med.

[5] Comprehensive genomic characterization of head and neck squamous cell carcinomas. Nature. 2015;517:576–82. 10.1038/nature14129 PMC4311405 25631445

[6] Sinevici N, O'Sullivan J (2016). Oral cancer: deregulated molecular events and their use as biomarkers. Oral Oncol.

[7] Mantovani F, Walerych D, Sal GD. Targeting mutant p53 in cancer: a long road to precision therapy. FEBS J. 2017;284:837–50. 10.1111/febs.13948 27808469

[8] Vogiatzi F, Brandt DT, Schneikert J, Fuchs J, Grikscheit K, Wanzel M (2016). Mutant p53 promotes tumor progression and metastasis by the endoplasmic reticulum UDPase ENTPD5. Proc Natl Acad Sci U S A.

[9] Belletti B, Baldassarre G (2011). Stathmin: a protein with many tasks. New biomarker and potential target in cancer. Expert Opin Ther Targets.

[10] Gigant B, Curmi PA, Martin-Barbey C, Charbaut E, Lachkar S, Lebeau L (2000). The 4 a X-ray structure of a tubulin:stathmin-like domain complex. Cell.

[11] Hsieh SY, Huang SF, Yu MC, Yeh TS, Chen TC, Lin YJ (2010). Stathmin1 overexpression associated with polyploidy, tumor-cell invasion, early recurrence, and poor prognosis in human hepatoma. Mol Carcinog.

[12] Jeon TY, Han ME, Lee YW, Lee YS, Kim GH, Song GA (2010). Overexpression of stathmin1 in the diffuse type of gastric cancer and its roles in proliferation and migration of gastric cancer cells. Br J Cancer.

[13] Belmont LD, Mitchison TJ (1996). Identification of a protein that interacts with tubulin dimers and increases the catastrophe rate of microtubules. Cell.

[14] Ngo TT, Peng T, Liang XJ, Akeju O, Pastorino S, Zhang W (2007). The 1p-encoded protein stathmin and resistance of malignant gliomas to nitrosoureas. J Natl Cancer Inst.

[15] Howitt BE, Nucci MR, Drapkin R, Crum CP, Hirsch MS (2013). Stathmin-1 expression as a complement to p16 helps identify high-grade cervical intraepithelial neoplasia with increased specificity. Am J Surg Pathol.

[16] Singer S, Malz M, Herpel E, Warth A, Bissinger M, Keith M (2009). Coordinated expression of stathmin family members by far upstream sequence element-binding protein-1 increases motility in non-small cell lung cancer. Cancer Res.

[17] Cheng AL, Huang WG, Chen ZC, Peng F, Zhang PF, Li MY (2008). Identification of novel nasopharyngeal carcinoma biomarkers by laser capture microdissection and proteomic analysis. Clin Cancer Res.

[18] Chen J, Abi-Daoud M, Wang A, Yang X, Zhang X, Feilotter HE (2013). Stathmin 1 is a potential novel oncogene in melanoma. Oncogene.

[19] Ma HL, Jin SF, Tao WJ, Zhang ML, Zhang ZY (2016). Overexpression of stathmin/oncoprotein 18 correlates with poorer prognosis and interacts with p53 in oral squamous cell carcinoma. J Craniomaxillofac Surg.

[20] Murphy M, Ahn J, Walker KK, Hoffman WH, Evans RM, Levine AJ (1999). Transcriptional repression by wild-type p53 utilizes histone deacetylases, mediated by interaction with mSin3a. Genes Dev.

[21] Singer S, Ehemann V, Brauckhoff A, Keith M, Vreden S, Schirmacher P (2007). Protumorigenic overexpression of stathmin/Op18 by gain-of-function mutation in p53 in human hepatocarcinogenesis. Hepatology.

[22] Chen WQ, Kang SU, Lubec G (2006). Protein profiling by the combination of two independent mass spectrometry techniques. Nat Protoc.

[23] Tanaka C, Uzawa K, Shibahara T, Yokoe H, Noma H, Tanzawa H (2003). Expression of an inhibitor of apoptosis, survivin, in oral carcinogenesis. J Dent Res.

[24] Zhong LP, Pan HY, Zhou XJ, Ye DX, Zhang L, Yang X (2008). Characteristics of a cancerous cell line, HIOEC-B(a)P-96, induced by benzo(a)pyrene from human immortalized oral epithelial cell line. Arch Oral Biol.

[25] Song X, Xia R, Li J, Long Z, Ren H, Chen W (2014). Common and complex Notch1 mutations in Chinese oral squamous cell carcinoma. Clin Cancer Res.

[26] Ma HL, Yu C, Liu Y, Tan YR, Qiao JK, Yang X (2015). Decreased expression of glutathione S-transferase pi correlates with poorly differentiated grade in patients with oral squamous cell carcinoma. J Oral Pathol Med.

[27] Wasserman WW, Sandelin A (2004). Applied bioinformatics for the identification of regulatory elements. Nat Rev Genet.

[28] Messeguer X, Escudero R, Farre D, Nunez O, Martinez J, Alba MM (2002). PROMO: detection of known transcription regulatory elements using species-tailored searches. Bioinformatics.

[29] Farre D, Roset R, Huerta M, Adsuara JE, Rosello L, Alba MM (2003). Identification of patterns in biological sequences at the ALGGEN server: PROMO and MALGEN. Nucleic Acids Res.

[30] Yu X, Vazquez A, Levine AJ, Carpizo DR (2012). Allele-specific p53 mutant reactivation. Cancer Cell.

[31] Zhong LP, Zhang L, Yang X, Pan HY, Zhou XJ, Wei KJ (2009). Comparative proteomic analysis of differentially expressed proteins in an in vitro cellular carcinogenesis model of oral squamous cell carcinoma. Proteomics Clin Appl.

[32] Hafkamp HC, Speel EJ, Haesevoets A, Bot FJ, Dinjens WN, Ramaekers FC (2003). A subset of head and neck squamous cell carcinomas exhibits integration of HPV 16/18 DNA and overexpression of p16INK4A and p53 in the absence of mutations in p53 exons 5-8. Int J Cancer.

[33] Taylor D, Koch WM, Zahurak M, Shah K, Sidransky D, Westra WH (1999). Immunohistochemical detection of p53 protein accumulation in head and neck cancer: correlation with p53 gene alterations. Hum Pathol.

[34] Jadersten M, Saft L, Smith A, Kulasekararaj A, Pomplun S, Gohring G (2011). TP53 mutations in low-risk myelodysplastic syndromes with del(5q) predict disease progression. J Clin Oncol.

[35] Min BM, Baek JH, Shin KH, Gujuluva CN, Cherrick HM, and Park NH. Inactivation of the p53 gene by either mutation or HPV infection is extremely frequent in human oral squamous cell carcinoma cell lines. Eur J Cancer B Oral Oncol 1994;30b:338–45. 10.1016/0964-1955(94)90036-1 7703804

[36] Ferguson BE, Oh DH (2005). Proficient global nucleotide excision repair in human keratinocytes but not in fibroblasts deficient in p53. Cancer Res.

[37] Zhou G, Wang J, Zhao M, Xie TX, Tanaka N, Sano D (2014). Gain-of-function mutant p53 promotes cell growth and cancer cell metabolism via inhibition of AMPK activation. Mol Cell.

[38] Wang F, Wang LX, Li SL, Li K, He W, Liu HT (2011). Downregulation of stathmin is involved in malignant phenotype reversion and cell apoptosis in esophageal squamous cell carcinoma. J Surg Oncol.

[39] Alli E, Yang JM, Hait WN (2007). Silencing of stathmin induces tumor-suppressor function in breast cancer cell lines harboring mutant p53. Oncogene.

[40] Wang J, Yao Y, Ming Y, Shen S, Wu N, Liu J (2016). Downregulation of stathmin 1 in human gallbladder carcinoma inhibits tumor growth in vitro and in vivo. Sci Rep.

[41] Walerych D, Lisek K, Del Sal G (2015). Mutant p53: one, no one, and one hundred thousand. Front Oncol.

[42] Candeias MM, Hagiwara M, Matsuda M (2016). Cancer-specific mutations in p53 induce the translation of Delta160p53 promoting tumorigenesis. EMBO Rep.

[43] Carney BK, Cassimeris L (2010). Stathmin/oncoprotein 18, a microtubule regulatory protein, is required for survival of both normal and cancer cell lines lacking the tumor suppressor, p53. Cancer Biol Ther.

[44] Sonego M, Schiappacassi M, Lovisa S, Dall'Acqua A, Bagnoli M, Lovat F (2013). Stathmin regulates mutant p53 stability and transcriptional activity in ovarian cancer. EMBO Mol Med.

[45] Johnsen JI, Aurelio ON, Kwaja Z, Jorgensen GE, Pellegata NS, Plattner R (2000). p53-mediated negative regulation of stathmin/Op18 expression is associated with G(2)/M cell-cycle arrest. Int J Cancer.

