# Supplementary figures and images for: Stathmin is overexpressed and regulated by mutant p53 in oral squamous cell carcinoma
Source: J Exp Clin Cancer Res. 2017 Aug 14;36:109. doi: 10.1186/s13046-017-0575-4 (PMC5556353; doi:10.1186/s13046-017-0575-4)

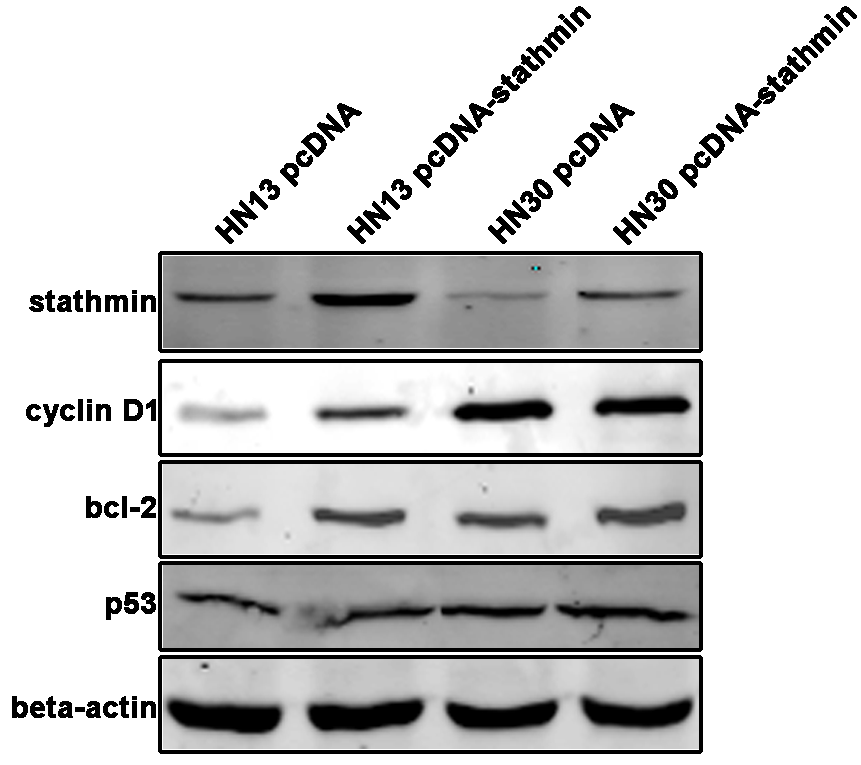

Supplement: Supplementary file 1 — In HN13 and HN30 cells transfected with stathmin overexpression vector, increased stathmin, cyclin D1, and bcl-2 expression were found when compared to those transfected with pcDNA, while no change in p53 expression. (TIFF 139 kb) [file 13046_2017_575_MOESM1_ESM.tif]

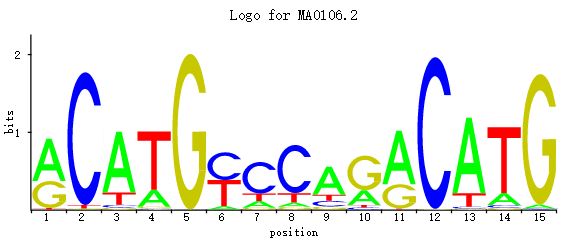

Supplement: Supplementary file 2 — The binding motif of p53 was predicted, utilizing data publically available on JASPAR (http://jaspar.genereg.net/). (JPEG 30 kb) [file 13046_2017_575_MOESM2_ESM.jpg]
